# Surgical margins of the oral cavity: is 5 mm really necessary?

**DOI:** 10.1186/s40463-022-00584-8

**Published:** 2022-10-04

**Authors:** James Fowler, Yael Campanile, Andrew Warner, Francisco Laxague, Naif Fnais, Kevin Fung, Adrian Mendez, Danielle MacNeil, John Yoo, David Palma, Anthony Nichols

**Affiliations:** 1 Department of Otolaryngology – Head and Neck Surgery, Victoria Hospital, London Health Science Centre, Western University, Room B3-431A, 800 Commissioners Road East, London, ON N6A 5W9 Canada; 2 Western University, London, ON Canada; 3 Department of Radiation Oncology, Western University, London, ON Canada

**Keywords:** Margin, Squamous cell, Carcinoma, Oral cavity

## Abstract

**Background:**

Squamous cell carcinoma is the most common malignancy of the oral cavity. Primary treatment involves surgical resection of the tumour with a surrounding margin. Historically, the most commonly accepted margin clearance is 5 mm. This distance is controversial, with recent publications suggesting closer margins do not impact local recurrence and survival. The objective of this study is to determine the closest surgical margin that does not impact local recurrence and overall survival.

**Methods:**

A retrospective review of the London Health Sciences Centre Head and Neck Multidisciplinary Clinic between 2010 and 2018 was performed. Demographic data, subsite, tumour staging, treatment modality, margins, and survival outcomes were analyzed. The primary endpoint was local recurrence free survival. Secondary endpoints included recurrence-free survival and overall survival. Descriptive statistics, as well as univariable and multivariable Cox proportional hazards regression modelling were performed for all patients.

**Results:**

Four-hundred and twelve patients were included in the study, with a median follow-up of 3.3 years. On univariable analysis, positive margins and margins < 1 mm were associated with significantly worse local recurrence-free survival, recurrence-free survival, and overall survival (*p* < 0.05), compared to margins > 5 mm. Patients with surgical margins > 1 mm experienced similar outcomes to those with margins > 5 mm. Multivariable analysis identified age of diagnosis, alcohol consumption, pathological tumour and nodal category as predictors of local recurrence free survival.

**Conclusions:**

Although historical margins for head and neck surgery are 5 mm, similar outcomes were observed for margins greater than 1 mm in our cohort. These findings require validation through multi-institutional collaborative efforts.

**Graphical abstract:**

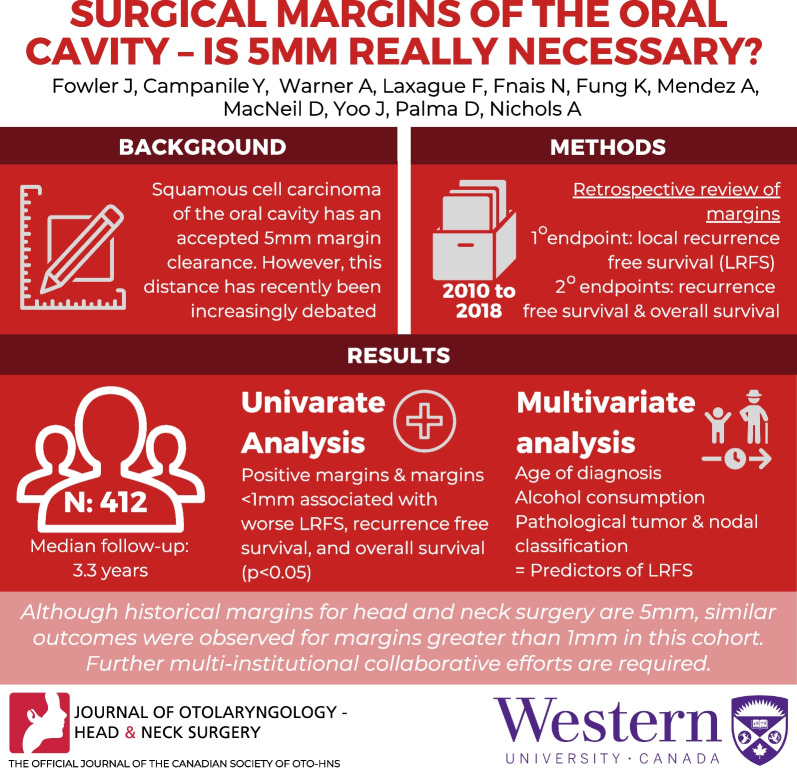

## Background

In recent years, the incidence of oral cavity cancer (OCC) has slowly been increasing. Globally, cancers of the oral cavity represent the 16th most common cancer [1], and is the most common non-cutaneous malignancy of the head and neck [2]. In 2020, there were approximately 377,000 new diagnoses of OCC worldwide, and 178,000 associated deaths [3]. Squamous cell carcinoma accounts for greater than 90% of these cases [4, 5], with the oral tongue and floor of mouth being the most common sites. [2]

Treatment of oral cavity squamous cell carcinoma (OCSCC) involves complete surgical resection of the tumour with adequate surrounding margins. Commonly this is followed by radiation with or without chemotherapy, when indicated. Overall survival, rate of recurrence, and need for adjuvant therapy is greatly dependant on resection of the tumour with margins clear of invasive carcinoma [6–8]. Historically, 5 mm of surrounding tissue not involved with cancer is considered a “clear” margin [9]. However, 5 mm margins are not always easily achievable due anatomical constraints and tissue contracture with formalin fixation. Depending on the surgical site and tumour stage, tissue contracture can result in a 20–50% reduction in margin distance [10, 11]. Attainment of these historical margins requires a larger specimen to be taken intra-operatively to compensate for the associated contracture, thus resulting in larger defects and increased patient morbidity.

Margin status is controversial within the literature, with recent studies suggesting lesser margins do not significantly impact local recurrence and overall survival [12–17]. At the London Health Sciences Centre (London, Ontario, Canada) we have historically defined clear margins as 3 mm or greater. The purpose of the present study is to determine the closest surgical margin that does not significantly impact local recurrence and overall survival for patients with oral cavity cancer.

## Methods

Ethics approval was obtained for the study (HSREB 7182). Retrospective review of patients seen in the London Health Sciences Centre (LHSC) Head and Neck Multidisciplinary Clinic between 2010 and 2018 was carried out. All patients diagnosed with OCSCC and underwent surgical resection (± adjuvant therapy) were included in the study. Patients with multiple primaries, or previous head and neck cancers were excluded. Patient data including demographics, tumour subsite, staging, treatment modality, surgical margins, recurrence, and overall survival were all collected and analysed. The primary endpoint for the study was local recurrence free survival (LRFS), while overall survival (OS) and recurrence free survival (RFS) were both secondary endpoints.

All statistical analysis was performed using SAS version 9.4 software (SAS Institute, Cary, NC, USA). Descriptive statistics were generated for all patient variables. Univariable and multivariable Cox proportional hazards regression were performed for all survival end points. All models were evaluated using Harrell’s concordance and integrated time-dependent area under the curve values. All eligible variables were incorporated into a multivariable regression model and sequentially removed using backward elimination techniques until all remaining covariates had *p*-values < 0.05. Kaplan–Meier estimates were generated for all survival end points, stratified by margin depth (mm), and compared using the log-rank test.

## Results

A total of 412 patients were included in the analysis. The median follow-up duration was 3.3 years.

### Patient demographics

Detailed patient demographics are shown in Table 1. The average age of diagnosis was 63 years old, with the majority of the patients being male (68%). Most patients had good overall performance scores with 90% of patients having an Eastern Cooperative Oncology Group (ECOG) scores of 0 or 1. With regards to smoking, 77% of patients were current or previous smokers, and the average number of pack years was 35. Alcohol abuse was common, with 37% of patients consuming more than 20 drinks per week.

**Table 1:** Patient demographics

Variable	N	Relative frequency
Age at diagnosis – mean ± SD,	412	63.7 ± 12.3
Male – *n*(%)	412	280 (68.0)
ECOG performance status – *n* (%)		
0 1 2 3 4	273	99 (36.3) 148 (54.2) 17 (6.2) 8 (2.9) 1 (0.4)
ECOG performance status – *n* (%)		
0–1 2–4	273	247 (90.5) 26 (9.5)
Smoking status – *n* (%)		
Current Previous Never	410	213 (52.0) 101 (24.6) 96 (23.4)
Smoking pack-years – mean ± SD	295	35.6 ± 21.6
Alcohol misuse – *n* (%)		
Current Previous Never	396	110 (27.8) 37 (9.3) 249 (62.9)
Alcohol per week – mean ± SD	339	12.6 ± 19.6
Immunosuppression – *n* (%)	412	8 (1.9)
Previous non-head-and-neck cancer – *n* (%)	412	47 (11.4)

### Tumour characteristics and treatment modality

The most common subunits of the oral cavity involved were the oral tongue (47%), floor of mouth (17%), and mandibular alveolus (13%). The majority of patients presented with early T1/T2 disease (58%), while T3 and T4 disease accounted for 12% and 29%, respectively. Clinically, no nodes were appreciated on imaging or physical exam preoperatively in 69% of patients. At the time of primary tumour resection, 75% of patient underwent neck dissection. Sixty-five percent of neck dissections were unilateral. Post-resection, forty-five percent of patients underwent adjuvant radiation therapy, and an additional 19% received chemotherapy as well (Table 2).

**Table 2:** Tumour characteristics and treatment modality

Variable	N	Relative frequency
Oral cavity subsite – *n* (%)		
Oral tongue	412	193 (46.8)
Floor of mouth		68 (16.5)
Mandible alveolus		52 (12.6)
Mandible alveolus		26 (6.3)
Retromolar trigone		26 (6.3)
Buccal		24 (5.8)
Other		23 (5.6)
Clinical *T* stage – *n* (%)		
T1	412	110 (26.7)
T2		130 (31.6)
T3		51 (12.4)
T4		121 (29.4)
Clinical N stage – *n* ( %)		
N0	412	284 (68.9)
N1		48 (11.7)
N2		79 (19.2)
N3		1 (0.2)
Neck dissection – *n* (%)	412	310 (75.2)
Regional neck surgery laterality – *n* (%)		
Bilateral	310	109 (35.2)
Unilateral		201 (64.8)
Adjuvant Therapy		
None – *n* (%)	412	146 (35.4)
Radiotherapy – *n* (%)		187 (45.4)
Chemoradiotherapy – *n* (%)		79 (19.2)

### Surgical margins

On final pathology, the average margin distance was 2.70 ± 2.44 mm. Nineteen percent of specimen margins were positive, and 24% were within 1 mm of invasive carcinoma (Table 3).

**Table 3:** Surgical margin characteristics

Variable	N	Relative frequency
Closest margin (mm) – mean ± SD, median	401	2.70 ± 2.44
Closest margin (mm) – *n* (%)		
0 (positive)	401	74 (18.5)
> 0, ≤ 1		95 (23.7)
> 1, ≤ 2		43 (10.7)
> 2, ≤ 3		65 (16.2)
> 3, ≤ 5		73 (18.2)
> 5		51 (12.7)
Positive patient margins – *n* (%)	408	79 (19.4)
Re-excision of suspected close margin– *n* (%)	412	73 (17.7)
Re-excision positive margin status – *n* (%)	73	14 (19.2)

### Univariable analysis

**Fig. 1:**
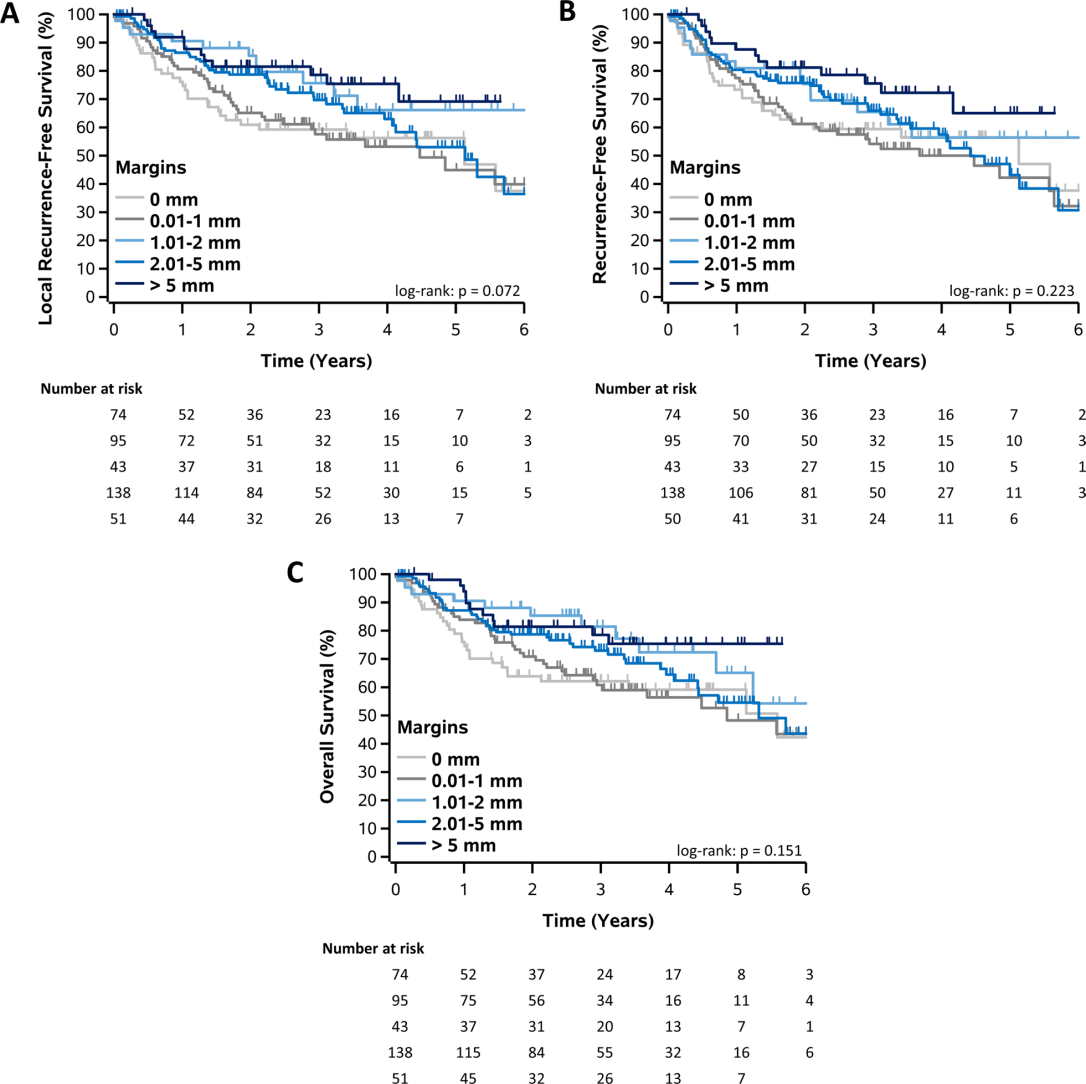
Kaplan–Meier curves for each survival end-point. Local recurrence free survival (**A**), recurrence free survival (**B**), overall survival (**C**)

Compared to margins > 5 mm, positive margins (*p* = 0.03) and margins < 1 mm (*p* = 0.03) had significantly poorer LRFS. This correlates with a hazard ratio of 2.15 for positive margins (95% CI, 1.10–4.18), and 2.01 for margins < 1 mm (95%CI, 1.05–3.83). Patients with surgical margins > 1 mm experienced similar outcomes to those with margins > 5 mm. Like LRFS, a positive margin or a margin < 1 mm resulted in poorer RFS (*p* = 0.04 and 0.04, respectively) and OS (*p* = 0.03 and 0.05, respectively). Figure 1 illustrates Kaplan–Meier curves for each survival end-point.

### Multivariable analysis

**Fig. 2:**
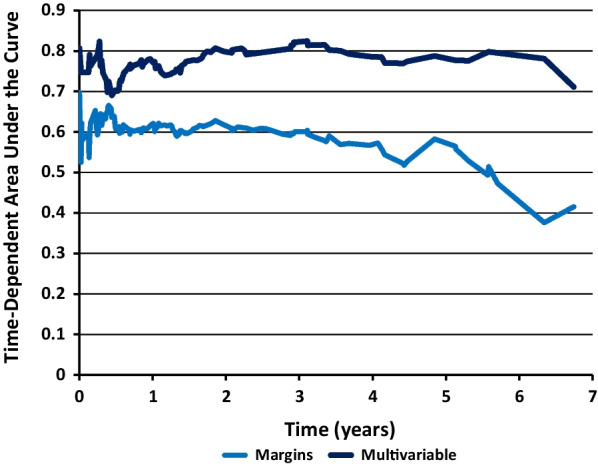
Time-dependent area under the curve plot for local recurrence-free survival comparing margins (0 mm, 0.01–1 mm, 1.01–2 mm, 2.01–5 mm, vs. > 5 mm) and multivariable model (adjusting for age, alcohol use and pathological *T* and *N* stage)

Strongest predictors of LRFS were age of diagnosis (*p* =  < 0.001), pathologic tumour stage (*p* = 0.003), pathologic nodal stage (*p* =  < 0.001), and alcohol consumption per week (*p* =  < 0.001). Surgical margins were not retained on multivariable analysis. Summary of results are available in Table 4. Time-dependent area under the curve plot for LRFS comparing multivariable analysis and margin status is depicted in Fig. 2.

**Table 4:** Multivariable analysis – predictive factors associated with local recurrence free survival

Multivariable analysis—local recurrence free survival		
Variable	Hazard Ratio (95% CI)	*P*-value
Age of diagnosis (per 5 years)	1.20 (1.09, 1.32)	< 0.001
Alcohol per week (per 10 drinks)	1.26 (1.16, 1.38)	< 0.001
Pathologic T stage (vs. T1)		0.003
T2	2.17 (1.20, 3.92)	0.010
T3-4	2.78 (1.55, 5.01)	< 0.001
Pathologic N stage (vs. N0)		< 0.001
N1	1.05 (0.51, 2.18)	0.887
N2-N3	2.95 (1.90, 4.58)	< 0.001

## Discussion

Although 5 mm has been considered the historic standard within the oral cavity, this is not based on level 1 evidence. Herein we present the largest Canadian study analysing surgical margins of the oral cavity. Our findings are consistent with other recent studies [12–17], which suggest 5 mm margins may not be necessary.

In a recent survey of the American Head and Neck Society, 56% of respondents classified a clear margin as > 5 mm on microscopic evaluation [18]. Although this consensus is held true for most head and neck surgeons, there has been a strong push within the literature to redefine the definition for clear margin. In a study by Zanoni et al., [14] surgical margins of 381 patients with OCSCC were analyzed. Their findings indicate that patients with surgical margins ≤ 2.2 mm had significantly poorer LRFS, while those between 2.3 and 5.0 mm showed no significant difference. A similar investigation was conducted by Tasche et al. [15] In their retrospective review of 432 patients, they determined that there was a significant increased risk of recurrence with margins < 1 mm in close agreement with our study. Additional resection beyond 1 mm did not correspond to a significant difference in recurrence rates. Lastly, Bajwa et al [13] performed a retrospective, multicentre analysis (*n* = 669) assessing impact of surgical margins on LRFS, disease free survival (DFS), and OS. The results of their study revealed margins < 1 mm were associated with significantly poorer LRFS and DFS. These studies compliment the findings of the current paper well. Despite these findings, larger, multicentre analyses are still required for widespread adoption of new definition for clear margins. We are initiating such a multicentre study in collaboration with multiple other Canadian centres.

In the present study, multivariable analysis revealed age of diagnosis, tumour stage, pathologic nodal stage, and alcohol consumption per week were the strongest predictors of LRFS. Age of diagnosis, tumour staging, pathologic staging are all predictive factors that have been reported in previous studies [5, 6, 19]. Surprisingly, surgical margins were not retained on final analysis in our study. This finding contradicts the multivariable analysis of Bajwa et al. [13] and Zanoni et al. [14] We hypothesize that this may be due to lack of power. We will re-examine this as part of our future multicentre analysis.

Our study is not without limitations. Adjuvant therapy is a major confounding factor within our study, with 45% of patients undergoing radiation therapy post-operatively. Due to the retrospective design, it is not possible to determine the cause and effect relationship that this had on margin status, LRFS, RFS, and OS. Our study is also single centred and of modest sample size. Further multicentre analysis is required to validate our findings.

## Conclusions

Our study has shown that surgical margins < 1 mm were associated with poorer LRFS, OS, and RFS. Moreover, comparing surgical margins > 1 mm versus > 5 mm showed no significant difference for disease control. Lastly, our study identified predictive factors for LRFS, which included age of diagnosis, tumour stage, pathological nodal stage, and alcohol consumption per week. The future direction of this study will be in partnership with Collaborative Research Initiative of the Canadian Society of Otolaryngology. The existing database has collected surgical outcomes data from approximately five thousand of patients. We plan to utilize this dataset to examine margin status with markedly greater statistical power to strengthen our conclusions.

## Data Availability

All data generated or analysed during this study are included in this published article.

## Abbreviations

OCC: Oral cavity cancer
OCSCC: Oral cavity squamous cell carcinoma
LHSC: London health sciences centre
LRFS: Local recurrence free survival
OS: Overall survival
RFS: Recurrence free survival
ECOG: Eastern cooperative oncology group
DFS: Disease free survival
